# Paratubal borderline serous tumor in a postmenopausal woman: a case report

**DOI:** 10.11604/pamj.2019.32.129.18031

**Published:** 2019-03-18

**Authors:** JongChul Baek

**Affiliations:** 1 Department of Obstetrics and Gynecology, College of Medicine, Gyeongsang National University, Gyeongsang National University Changwon Hospital, Changwon, Republic of Korea

**Keywords:** Paratubal cyst, paratubal borderline serous tumor, menopause

## Abstract

Unlike borderline ovarian tumors, paratubal borderline tumors are extremely rare gynecologic tumors. They occur in reproductive-aged females at an earlier stage of disease and have a good prognosis. A 61-year-old woman, gravida 3 para 3, presented with progression of ovarian cyst. Computed tomography revealed a 6-cm simple cystic lesion without enhancing papillary projections. The patient underwent total laparoscopic hysterectomy and bilateral salpingo-oophorectomy. Frozen specimens showed that the tumor was benign, thereby confirming a final diagnosis of paratubal borderline serous tumor. The patient refused comprehensive surgical staging and opted for close follow-up. The patient remains asymptomatic with no evidence of recurrence at the 24-month follow-up. To the best of our knowledge, this is the first reported case of paratubal borderline serous tumor in a postmenopausal patient. The findings of this study and those of other case reports can contribute to the understanding, diagnosis, treatment and prognosis of these rare tumors.

## Introduction

In asymptomatic postmenopausal women, most adnexal cysts are benign and simple cysts with a prevalence of 15%-20%. Of these, paratubal cysts have a prevalence of 5% [1, 2]. Most cysts are asymptomatic and are found incidentally during pelvic surgery [3]. To date, nine cases of primary paratubal borderline tumors (PBTs) have been reported in reproductive-aged patients, of which seven were paratubal borderline serous tumors, one was a borderline mucinous tumor and one was a borderline endometroid tumor [3-5]. Here we present the eighth case of paratubal borderline serous tumor to be reported in the literature and the first to be reported in a postmenopausal woman. PBT is similar to ovarian cancer in histological appearance; however, its clinical course and prognosis are not well understood owing to its rarity. As there is currently no standard treatment for PBTs, treatment needs to be individualized. Fertility-sparing surgery should be considered in patients who wish to have children in the future and long-term follow-up is necessary to detect recurrence [3, 5]. In postmenopausal women, treatment has been extrapolated according to the guidelines for ovarian tumors, indicating that comprehensive surgical staging surgery may be preferred [6]. However, as there have been no reports of metastasis and positive lymph nodes in PBTs, it remains debatable whether comprehensive surgical staging surgery should be performed. Therefore, appropriate treatment guidelines for PBTs in postmenopausal patients should be developed based on the clinicopathological manifestation of these tumors reported in the literature. Here we report the case of a postmenopausal woman with PBTs who underwent hysterectomy and bilateral salpingo-oophorectomy (BSO), exhibiting no evidence of recurrence at the 24-month follow-up.

## Patient and observation

**Figure 1:**
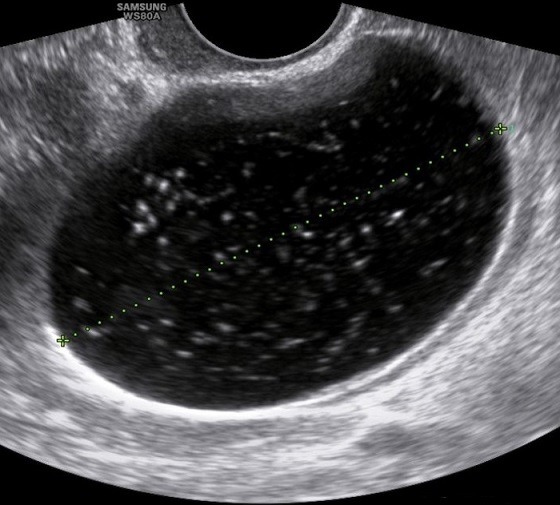
Transvaginal ultrasound image shows a low echogenic cystic mass with thin and smooth inner wall

**Figure 2:**
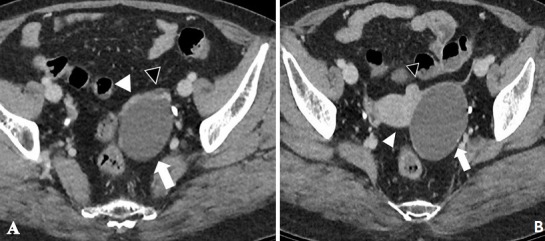
(A) contrast-enhanced CT images show a unilocular cystic lesion (white arrow) abutting the left ovary (black arrowhead) and fallopian tube (white arrowhead); (B) contrast-enhanced CT images show a unilocular cystic lesion (white arrow), uterus (white arrowhead) and myoma (black arrowhead)

**Figure 3:**
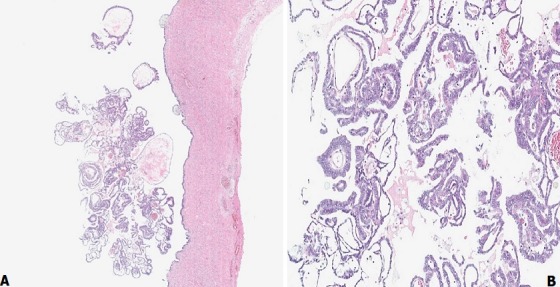
(A) polypoid lesion on the internal surface of the paratubal cyst (hematoxylin and eosin stain; original magnification × 20); (B) tumor cells show nuclear atypism and stratification without stromal invasion (hematoxylin & eosin stain; original magnification × 100)

**Figure 4:**
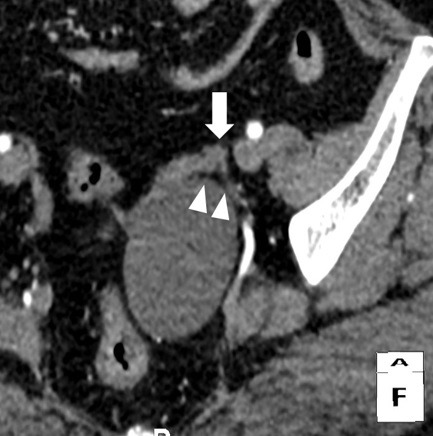
A CT multiplanar reconstruction (MPR) image shows linear fat planes (white arrowhead) between the left ovary (white arrow) and the cyst

A 61-year-old woman, gravida 3 para 3, presented with an increased size of ovarian cyst. Her history revealed a diagnosis of left ovarian cyst 3cm in diameter 3 years previously. Her serum cancer antigen (CA) 125 and CA 19-9 levels were within the normal ranges, and she was followed up at 6-month intervals at a private hospital. The ovarian cyst size had not changed in size and structure on biannual transvaginal ultrasound (TVS). However, she was referred to our institution in July 2016 with progression of ovarian cyst to 6cm on a pelvic ultrasonogram 2 weeks ago. The patient underwent menopause at the age of 48 years, with an uneventful past menstrual cycle. She had no medical history except the benign thyroid nodule, no relevant surgical history and had not received hormonal therapy. Routine blood chemistry and serum tumor marker analysis showed that the levels of carcinoembryonic antigen, CA 19-9, CA 125, and human epididymis protein 4 were all within the normal ranges. Uterine cervical cytology at the time of admission was normal. TVS showed a left ovarian unilocular cyst, size 6cm × 5cm × 4cm, with diffuse low-level internal echoes, thin walled, smooth margined, no septa and no papillary projections (Figure 1). The uterus size was 6cm × 4cm × 3cm, and a 1cm uterine myoma was found in the fundus. Pelvic contrast-enhanced computed tomography (ECT) images revealed a unilocular cystic lesion measuring 6 cm at its widest dimension, without enhancing solid intramural nodules and no evidence of lymphadenopathy or ascites (Figure 2). CT scan indicated benign ovarian cystic tumors. The patient underwent laparoendoscopic single-site (LESS) surgery. On laparoscopic inspection, the cyst was found to be located in the ampullary and fimbrial regions of the left fallopian tube. The left fallopian tube was grossly unremarkable and the left ovary was atrophied. The left ovary and tube were completely separate from the cyst and the cyst was surrounded by a translucent wall with serous cystic components, but solid components were not seen inside. Clinical diagnosis was suggestive of left paratubal cyst. The patient underwent hysterectomy and BSO. Although tumor markers were normal and there was no presence of papillary projections, the progression in size in the postmenopausal woman indicated a possible malignancy. Frozen sections were prepared from surgery specimens and confirmed a benign cyst. After surgery, the pelvic cavity was explored and no specific findings were found in the abdominal cavity. Histopathological examination of the paratubal cyst revealed the presence of polypoid lesions on the internal surface of the cyst. The tumor cells showed nuclear atypism and stratification, which are histological characteristics of borderline tumor (Figure 3). A final diagnosis of paratubal borderline serous tumor was confirmed. The patient was discharged from the hospital on postoperative day 3. CT images were retrospectively evaluated, focusing on the left ovary, fallopian tube, and paratubal cyst after surgery. Multiplanar reconstruction CT imaging revealed linear fat planes between the left ovary and the cyst (Figure 4), indicating that the left ovary, fallopian tube and paratubal cyst were separate structures, similar to those seen during surgery. The tumor was not completely staged, and a comprehensive surgical staging operation was recommended; however, the patient refused adjuvant surgical treatment and opted for close follow-up. Pelvic ECT, TVS and tumor markers were checked every three months. The patient remains asymptomatic and follow-up at 24 months showed no evidence of recurrence or metastasis.

## Discussion

Paratubal cysts are common disorders, representing approximately 10% of all adnexal masses [1, 7]. In asymptomatic postmenopausal women, the prevalence of adnexal cysts and paratubal cyst is reported to be 15% to 20% and 5%, respectively [2] and most adnexal cysts are benign and simple cysts. Unlike borderline ovarian tumors that account for 5% to 20% of all epithelial ovarian cancer, PBTs are extremely rare and are reported as case reports. Borderline tumors are identified by epithelial proliferation with no stromal invasion [8]. A MEDLINE search using the search terms “paratubal cyst, borderline tumor, low malignancy potential, and atypical proliferative tumors” revealed only nine published cases in English-language scientific literature since 2005. Of these nine previously reported cases of PBTs, there were seven borderline serous tumors, one borderline mucinous tumor and one borderline endometroid tumor (Table 1) [3-5, 7-12] and all cases occurred in premenopausal patients. The present case report of paratubal borderline serous tumor (PBST) is the eighth to be reported in the literature and the first in a postmenopausal woman. While paratubal cysts occur in all age groups, they are most common in the third and fourth decades of life [13]. Most of these cysts are asymptomatic and are usually discovered during routine examination or incidentally found during pelvic surgery. In symptomatic cases, dull unilateral pelvic pain is the most common symptom and surgery is necessary in cases of severe symptoms due to enlargement or torsion. PBTs is similar to ovarian cancer in histological appearance, but its clinical course and prognosis are not well understood [7, 8]. Most PBT cases have been reported to occur in reproductive-aged women, with an age range of 17-61 years with an earlier stage of disease [3]. In postpubertal years, an increase in the size of the paratubal cyst is observed due to the secretory activity of epithelium and the influence of hormonal activity [13]. Of previously reported cases, four of the ten presented with pelvic pain, two had menstrual abnormalities and four had asymptomatic adnexal cysts. Furthermore, of the ten patients, tumors were located on the right side in seven patients and on the left side in three patients. All tumors were unilateral, measuring 3-19cm in diameter. Tumors size appeared to be inversely proportional to the patient's age, with adolescent patients exhibiting larger sizes. Nine of the ten PBTs were described as unilocular and one was multilocular. Four patients underwent laparoscopic surgery. Various types of surgery were conducted depending on the patient's age, preference, and fertility preservation. All patients undergoing surgical staging were diagnosed using the FIGO staging of disease and showed a good prognosis without recurrence at follow-up periods.

**Table 1:** Paratubal borderline tumors reported in the literature

Author	Age (years)	Symptom/presentation	CA-125	Image/intraoperative finding	Surgery	Pathology (FIGO stage)	Follow-up
Salamon et al., 2005	45	Complex Rt adnexal mass	*	3 cm torsed, Rt cyst PP (USG)	Laparoscopic BO cystectomy and partial Rt salpingectomy Second, laparoscopic RSO	# Benign Endometriod BT	12 month NED
Seamon et al, 2009	26	RLQ pain and nausea	WNL	12.5 cm Rt simple cyst PP (OP finding)	Laparoscopic Rt paratubal cystectomy and partial salpingectomy FS-CSS (RSO, omentectomy, PPALND, appendectomy, multiple biopsies)	# BT Serous BT (Ⅰc)	12 month NED
Kumbak et al, 2010	39	Left adnexal mass in cesarean section	WNL	6 cm Lt cyst, PP (OP finding) 2 cm Lt cyst 2 cm Lt ovarian cyst (PET-CT)	Cesarean section and cyst extirpation Second, FS-CSS (cystectomy, omentectomy, PPALND, appendectomy) Third, Lt cystectomy, Rt ovarian wedge resection	# Not done Serous BT (Ⅰc) Benign cyst	15 month NED
Shin et al, 2011	27	Huge pelvic mass Flank pain	WNL	16 cm Rt cyst PP (pelvic ECT)	Exploratory laparotomy (cystectomy, multiple peritoneal biopsies, washing cytology)	# Serous BT (Ⅰa)	20 month NED
Terek et al, 2011	19	Abdominal pain (LLQ) nausea	WNL	10 cm torsed Lt cyst PP (USG)	Laparotomic cystectomy, peritoneal washings	# BT Serous BT	7 month NED
Im et al, 2011	20	Complex Rt adnexal mass	WNL	10 cm multilobuated Rt cyst PP (pelvic ECT)	Laparoscopic cyst enucleation FS-CSS (RSO, partial omentectomy, PLND, appendectomy)	# Mucinous BT (Ⅰa)	30 month NED
Kiseli et al., 2012	17	Menstrual irregularity Oligomenorreha	WNL	7cm unilocular complicated Cyst, PP (USG, pelvic MRI)	Laparotomic paratubal cystectomy, Rt ovarian wedge resection	# Benign Serous BT	12 month NED
Alaoui et al., 2012	38	RLQ pain, nausea, vomiting	WNL	10 cm twisted Rt cyst PP (OP finding)	Laparotomic paratubal cystectomy, Hysterectomy, BSO, omentectomy, washing, biopsies	# Not done Serous BT	12 month NED
Lee et al, 2014	17	Irregular menstruation Large Rt adnexal cyst	WNL	19 cm Rt unilocular cyst PP (USG, pelvic ECT)	LESS salpingectomy and Rt ovarian wedge resection	# Serous BT Serous BT	3 month NED
Baek et al, 2018	61	Progression of adnexal cyst	WNL	6 cm Lt simple cyst	LESS hysterectomy and BSO	# Benign cyst Serous BT	24 month NED

Abbreviations: Rt, Right; Lt, Left; *, Not specified; PP, papillary projections; USG, ultrasonography; BO, bilateral ovarian; RSO, right salpingo-oophorectomy; BSO, bilateral salpingo-oophorectomy; #, frozen biopsy; BT, borderline tumors; NED, no evidence of disease; RLQ, Right lower quadrant; WNL, within normal limits; OP, intraoperative; FS-CSS, fertility-sparing comprehensive surgical staging; PPALND, pelvic and para-aortic lymph node dissection; PLND, pelvic lymph node dissection; PET-CT, Positron emission tomography-computed tomography; ECT, enhanced computed tomography; LESS, laparoendoscopic single-site surgery

Nine of ten PBTs were described as having papillary projections on the image modalities or intraoperative findings, but only the present case showed no papillary projections. There were no cases of elevated serum CA 125 level in the ten cases and three of the eight frozen biopsy results were misdiagnosed as benign cysts. TVS is the most useful diagnostic tool to assess the risk of malignancy in adnexal masses and shows the high sensitivity but relatively low specificity in the diagnosis of paratubal cysts [1, 14]. While the risk of malignancy in unilocular adnexal cysts in postmenopausal women is low, some cases have reported contrasting conclusions [1], as measurement of serum CA125 did not significantly increase the diagnostic accuracy of TVS [15]. It is difficult to preoperatively diagnose PBTs due to the absence of typical ultrasound features, although observation of papillary projections on ultrasound is associated with an increased risk of malignancy [1, 14]. Papillary projections can also be seen in benign neoplastic conditions, such as paratubal cystadenoma and cystadenofibromas [1]. This makes it difficult to differentiate between malignant and benign tumors preoperatively. Without papillary projections, the risk of malignancy is low and suggestive of benign cysts. The nine previously reported cases all showed papillary projections in image studies, including TVS, GU-ECT and pelvic magnetic resonance imaging (MRI). TVS and GU-ECT images showed no papillary projection on the cyst, contrary to previously reported cases, which were preoperatively misdiagnosed as benign lesions in the present case. Progression in size of the cyst > 5cm or the presence of papillary projections may indicate borderline malignancy in postmenopausal patients. In all cases, including the present case, serum CA 125 levels were within the normal range. Tumor markers can be used to monitor recurrence of the disease rather than diagnose it. There is currently no standard treatment for PBTs owing to its rarity. Optimal treatment can be extrapolated according to the guidelines for ovarian tumors, as they have the same embryological origin. Patients wishing to preserve fertility may be amendable to fertility-sparing surgery. However, for patients who do not desire fertility preservation, comprehensive surgical staging surgery may be preferred (washing cytology, hysterectomy, BSO, pelvic-aortic lymphadenectomy, omentectomy, multiple peritoneal biopsies). Treatment should be individualized and long-term surveillance is important to detect recurrent disease after fertility-sparing surgery. TVS, serum CA 125 and pelvic ECT are commonly used during follow-up examinations. While there have only been a few published reports, no recurrence and metastasis have been reported in patients with PBTs. Pelvic and aortic lymphadenectomy is a controversial subject in ovarian borderline malignancy. There have been no reports of metastasis and positive lymph nodes in PBTs. Thus, it remains controversial whether comprehensive surgical staging surgery in perimenopause and postmenopausal patients should be performed. Appropriate treatment guidelines of PBTs in these patients need to be developed based on the clinical manifestation of these tumors by collecting data accumulated through case reports.

## Conclusion

PBSTs can occur in postmenopausal women without papillary projections on image studies and intraoperative findings. Reports of these rare tumors need to be shared to better understand diagnoses, treatments, prognoses, recurrences and metastases using data accumulated through case reports. Our findings contribute to the literature on PBT treatments and further long-term follow-up and additional case reports are required to elucidate the clinicopathological characteristics of the PBSTs.

## Competing interests

The author declare no competing interests.
